# Poorly differentiated adenocarcinoma with signet-ring cells in duodenal papilla: a case report

**DOI:** 10.1186/s40792-017-0287-1

**Published:** 2017-01-17

**Authors:** Yuta Ushida, Kiyoshi Hiramatsu, Satomi Saeki, Takeshi Amemiya, Hidenari Goto, Toshiyuki Arai

**Affiliations:** Department of Surgery, Anjo Kosei Hospital, 28 Higashihirokute, Anjo-cho, Anjo, Aichi 446-8602 Japan

**Keywords:** Duodenal papillary cholangiocarcinoma, Poorly differentiated adenocarcinoma, Signet-ring cell

## Abstract

An 82-year-old woman with common bile duct (CBD) dilatation observed during routine ultrasonography was referred to our hospital. Preliminary blood tests revealed elevated levels of hepatobiliary enzymes. Computed tomography (CT) scan showed lower bile duct wall thickening and enhancement. Esophagogastroduodenoscopy revealed mildly swollen papilla of Vater, without ulceration. Endoscopic retrograde cholangiography demonstrated that the CBD was grossly dilated with a constriction in the lower part. The final diagnosis indicated poorly differentiated adenocarcinoma of duodenal papilla with signet-ring cells; pT3N0M0, *stage IIA* (*Unio Internationalis Contra Cancrum*, 7th edition), for which subtotal stomach-preserving pancreaticoduodenectomy (SSPPD) was performed. This case is quite rare, and the surgery resulted in a desirable outcome. The patient has been disease-free for 5 years since the surgery.

## Background

Most duodenal papillary carcinomas (DPCs) are well differentiated [1]. Poorly differentiated DPCs are rare and have unfavorable prognosis. Signet-ring cells in the duodenal papilla are an indication of poor prognosis. Here, we report a case of poorly differentiated DPC with signet-ring cells that had favorable outcome.

## Case presentation

An 82-year-old woman with common bile duct (CBD) dilatation, as observed during routine ultrasonography, was referred to our hospital. She was asymptomatic. Laboratory test results were aspartate aminotransferase level, 278 IU/L; alanine aminotransferase level, 184 IU/L; alkaline phosphatase level, 1877 IU/L; total bilirubin level, 0.42 mg/dL; amylase level, 47 IU/L; and presence of routine inflammatory markers. Laboratory tumor marker levels, carcinoembryonic antigen, and carbohydrate antigen 19–9 levels were normal.

**Fig. 1:**
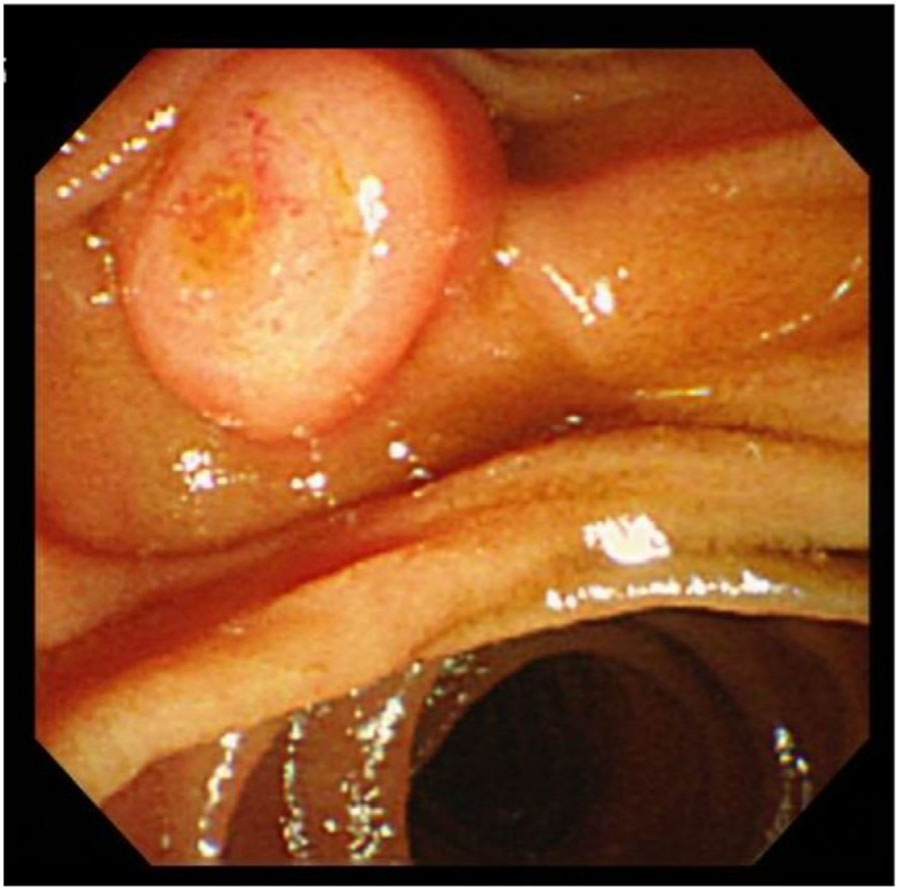
EGD reveals mildly swollen and protruded papilla of Vater, with no mucosal erosion. Cancer cells are not found in the biopsy specimen

Esophagogastroduodenoscopy (EGD) revealed mildly swollen papilla of Vater, without any mucosal erosion (Fig. 1).

**Fig. 2:**
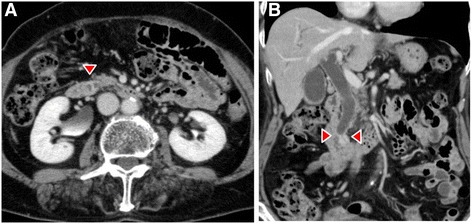
Dynamic CT images, arterial phase, **a** axial view, and **b** coronal view show thickening of the wall that is enhanced in the lower part of the CBD (*red arrowheads*), accompanied by a proximal dilatation of the biliary tract. No lymph node metastases are observed

**Fig. 3:**
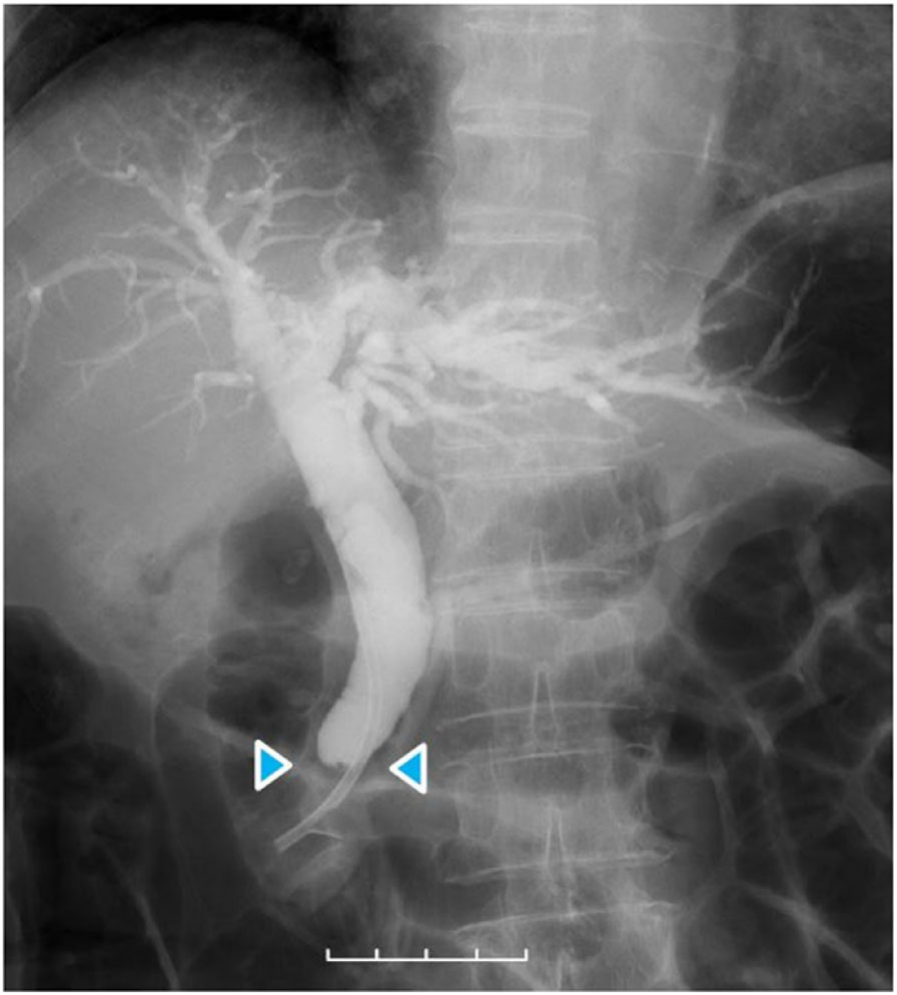
Cholangiography demonstrates that CBD was abruptly obstructed in the lower part (*blue arrowheads*) and was grossly dilated in the proximal part

**Fig. 4:**
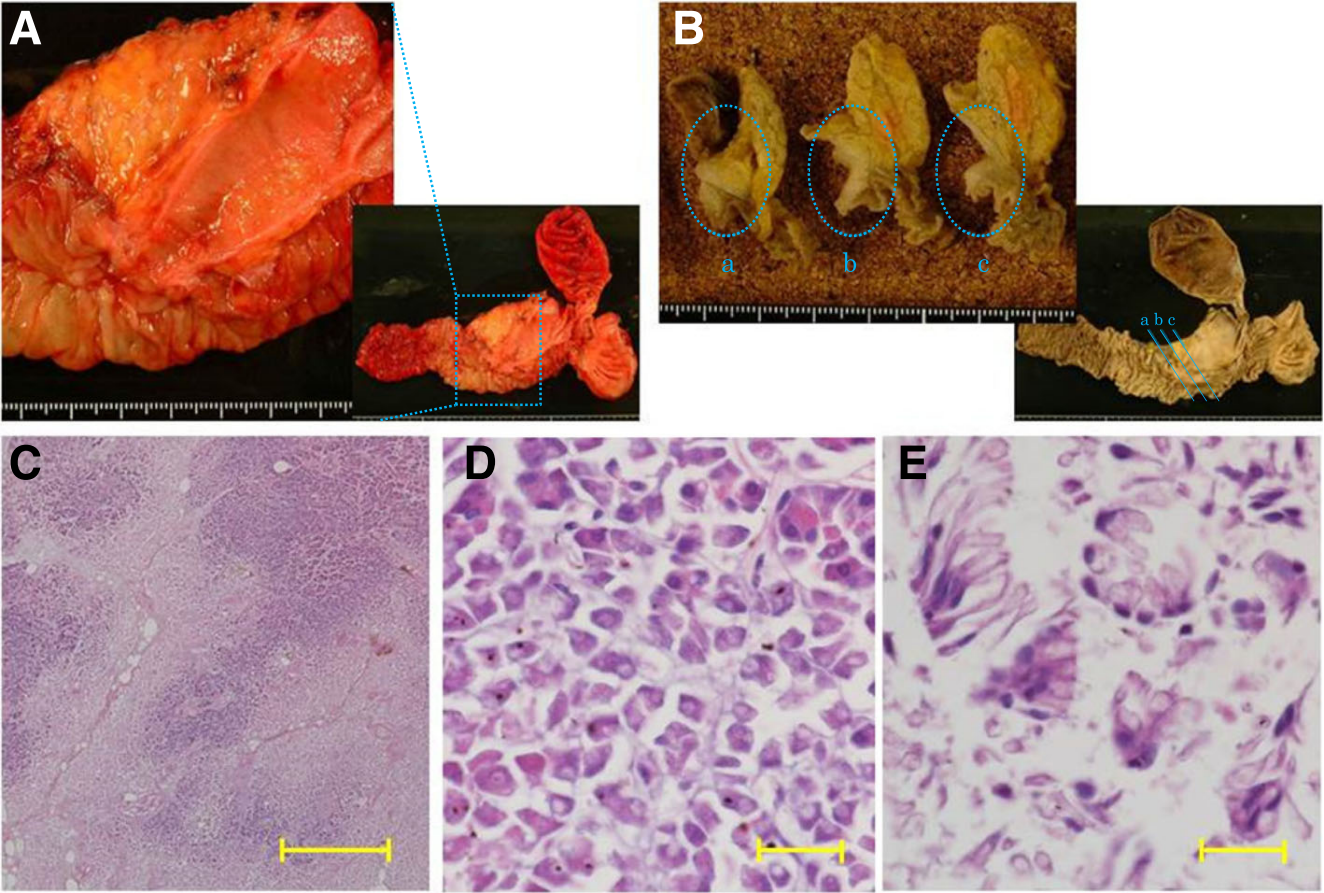
**a** Resected specimen. Macroscopically, there is an exposed protruded tumor (*interrupted square*). **b** The cut surface of the duodenal wall and the pancreatic head (**a** most anal; **c** most oral). From **a** to **c**, there are whitish tumors (*interrupted circles*). The *scale divisions* represent 1 mm each in **a** and **b**. **c** Histopathologically, the cancer cells show the pancreatic invasion with infiltrative growth and fibrosis. The most parts of the tumor are poorly differentiated adenocarcinoma, and the ratio of signet-ring cells was about 10–15% (hematoxylin-eosin, ×40, the *scale bars* indicate 1 mm). **d**, **e** Tumor cells contain mucin, which makes the nucleoli crescent-shaped (hematoxylin-eosin, ×600, the *scale bars* indicate 20 μm)

Dynamic computed tomography (CT) showed bile duct wall thickening that was enhanced in the lower part of the CBD (Fig. 2). Endoscopic retrograde cholangiography (ERC) demonstrated abrupt obstruction of the lower CBD (Fig. 3). Histological examination of biopsy specimens from the lower CBD showed adenocarcinoma with signet-ring cells. We diagnosed extrahepatic cholangiocarcinoma and performed subtotal stomach-preserving pancreaticoduodenectomy (SSPPD). The surgery lasted 348 min, and the total blood loss was 525 mL. Histopathology report suggested atypical epithelial cells exhibiting infiltrative growth, with fibrosis of the duodenal papilla (Fig. 4c). Tumor cells displayed intracytoplasmic mucus deposition, crescent-shaped nucleoli (Fig. 4d, e) extensions along the lower CBD, and invasion of pancreatic parenchyma (3 mm). AcbBd, exposed protruded type, 22 × 16 mm, por2/sig, pT3a, sci, INFc, ly1, v1, ne1, pN0, pHM0, pPM0, pEM0, PV0, A0, R0, pStage IIA according to the Japanese Classification on Cancer of the biliary tract [2] and pT3N0M0 stage IIA in accordance with Union for Internationatinal Cancer Control, 7th edition. The final diagnosis was poorly differentiated adenocarcinoma with signet-ring cell of DPC. The patient developed pancreatic fistula postoperatively (ISGPF grade B), with no other complications and was discharged on day 37 postsurgery. She has remained disease-free for 5 years since the surgery.

## Discussions

DPC is a rare clinical entity, occurring in less than 6 per million people annually. It represents 0.2% of all gastrointestinal cancers and accounts for only 6% of all cancers developing in the periampullary region [3]. The Japanese Society of Hepato-Biliary-Pancreatic Surgery reported that the most common histological type of DPC is well-differentiated adenocarcinoma (36.3%), followed by papillary adenocarcinoma (27.6%) and moderately differentiated adenocarcinoma (25.0%). Poorly differentiated adenocarcinoma is rare and accounts for 5.5% of all DPC cases [4]. Signet-ring cells are also extremely uncommon histologic types at this site and arise mainly from the stomach. Signet-ring cell carcinomas (SRCC) are characterized by signet-ring cells with intracytoplasmic mucin occupying more than 50% of the tumor [5]. In our case, the proportion of signet-ring cells to whole carcinoma was about 10–15%, so we diagnosed poorly differentiated adenocarcinoma with signet-ring cells, and not SRCC. Furthermore, poorly differentiated DPCs are rare, and on a PubMed search using key words like Vater, poorly differentiated, and signet, only ten well-documented cases were found (Table 1) [6–15]. The 11 cases, including our case, consisted of three men and eight women with ages ranging from 43 to 83 years (mean 62 years). Jaundice was the most common symptom (54.5%). Two cases of DPC with jaundice survived for more than 5 years after surgery, while rapid metastasis [13] was observed in others, as was disseminating carcinomatosis without jaundice [6].

**Table 1:** Published cases of poorly differentiated adenocarcinoma in duodenal papilla with signet-ring cells

Author	Year	Age (years)	Sex	Complaint	TNM stage^a^	Distant metastasis	Treatment	Histology	Follow-up (months)	Outcome
Nabeshima [5]	2003	49	M	Purpura (DIC^b^)	T3NxM1 stage IV	Lung, bone marrow	Chemotherapy	Por/sig	12	Died
Eriguchi[6]	2003	83	M	Jaundice	T3N0M0 stage IIA	–	PD	Sig	12	Alive
Ramia [7]	2004	67	F	Jaundice	T2N0M0 stage IB	–	PD	Sig	12	Alive
Akatsu [8]	2007	43	F	Jaundice	T2N0M0 stage IB	–	PD	Sig	90	Alive
Bloomston [9]	2006	58	F	Jaundice	T2N0M0 stage IB	–	PD	Sig	134	Alive
Ishibashi [10]	2009	59	M	Abdominal pain	T3N0M0 stage IIA	–	PD	Sig	18	Died
Ogata [11]	2010	42	F	Jaundice	T4N1M0 stage III	–	SSPPD	Sig	6	Alive
Matsuoka [12]	2013	61	F	Left visual disturbance	TxNxM1 stage IV	Brain	Gross total resection of brain tumor	Sig	3	Died
Acharya [13]	2013	78	F	Jaundice	T3N0M0 stage IIA	–	PD	Sig	6	Alive
Wakasugi [14]	2015	59	F	Elevated transaminase	T3N1M1 stage IV	Paraaortic node	PD	Sig	7	Alive
Our case	2016	82	F	–	T3N0M0 stage IIA	–	SSPPD	Por/sig	60	Alive

^a^International Union Against Cancer TNM classification

^b^Disseminated intravascular coagulation

The 5-year survival rate in patients with DPC after radical resection is 30–68% [16–19]. In most cases, the prognosis of DPC is better than biliary and pancreatic carcinomas. Owing to their anatomical location, ampullary tumors become clinically apparent earlier because of biliary or pancreatic duct occlusion. Since DPC is often diagnosed at an early stage, surgical resection has a higher probability of success. The average recurrence interval of DPC was 13–22 months [19, 20], and recurrence includes liver metastasis, local recurrence, peritoneal metastasis, and bone metastasis [19–21]. The important factors affecting the prognosis are lymph node status, depth of tumor invasion, and degree of tumor differentiation [22, 23]. Patients with pancreatic infiltration tend to have early recurrences [24]. Lymph node status is a significant predictive factor in liver metastasis, and a 5-year survival rate in patients with lymph node-positive status is 19.1% and of those with node-negative status is 63.7% [21]. Immunohistochemical staining patterns of cytokeratin and mucin allow further classification of SRCC to intestinal, pancreatobiliary, gastric, and mixed type. SRCC patients with intestinal type are favorable, and those with mixed type reveal poor prognosis.

In our case, although the tumor consisted of poorly differentiated adenocarcinoma with signet-ring cells and had infiltrated into the pancreatic parenchyma pT3, both of which indicating poor prognosis, the outcome was desirable. She was diagnosed with DPC incidentally in the health checkup before having jaundice; therefore, the ratio of signet-ring cells was relatively low. Similar reports are very few for DPC [25].

It is well known that surgical resection is the only curative treatment for DPC. No evidence-based chemotherapy regimens exist either for the treatment of unresectable cancers or for postoperative adjuvant therapy; therefore, we simply followed up the patient. Fortunately, the patient is doing well, without any signs of tumor recurrence since the last 5 years.

## Conclusions

Here, we have presented favorable results in a case of poorly differentiated DPC with signet-ring cells. It is however important to study some more cases with similar outcomes to establish its characteristics.

## Abbreviations

CBD: Common bile duct
CT: Computed tomography
DPC: Duodenal papilla carcinoma
EGD: Esophagogastroduodenoscopy
SRCC: Signet-ring cell carcinoma
SSPPD: Subtotal stomach-preserving pancreaticoduodenectomy
